# Hard-Headed Decisions: Intrapersonal Factors Underlying Concussion Reporting in University Athletes

**DOI:** 10.1089/neur.2023.0030

**Published:** 2023-08-17

**Authors:** William Archambault, Dave Ellemberg

**Affiliations:** ^1^ École de Kinésiologie et des science de l'activité physique, Université de Montréal, Montréal, Quebec, Canada.; ^2^ Ingram School of Nursing, McGill University, Montréal, Quebec, Canada.

**Keywords:** concussion disclosure, decision making, grounded theory, head injuries, sport concussion, qualitative interviews

## Abstract

Most of the research investigating sports concussion (SC) disclosure has been conducted using questionnaires with a pre-determined set of questions. Hence, significant gaps remain in our understanding of which factors weight in the decision-making process underlying SC disclosure and how they contribute to it. This present study aims to fill some of these gaps using qualitative methods to identify intrapersonal determinants of SC disclosure and describe their influence on an athlete's decision-making process. Our results are based on in-depth, semistructured interviews (range, 56–79 min; total = 587 min) with 9 university athletes (5 females, 4 males) from three team sports (soccer, rugby, and cheerleading). Using constant comparative analysis guided by Straussian grounded theory, we identified 13 concepts, across three major intrapersonal categories (i.e., attitudes and behaviors; concussion knowledge; and subjective evaluation of the concussion), contributing to SC disclosure, including novel determinants such as prioritization of athletic versus intellectual activities and maturity level. Our results suggest that a comparison between experiential knowledge and severity of the injury plays a major role in determining an athlete's disclosure behaviors. Athletes with a history of concussion seem to adopt a non-disclosure default strategy and are inclined to disclose their concussion symptoms only if they judge their current concussion to be worse than their previous most severe injury. Other concepts identified appear to contribute to the decisional process by modulating the adoption of this non-disclosure default strategy. Our work highlights the benefits and necessity of using qualitative methods to study the decision-making process underlying concussion disclosure.

## Introduction

Despite advances in clinical imaging and cognitive testing, the diagnosis of sports concussions (SCs) remains dependent on symptom disclosure.^1^ This is disquieting given that a significant proportion of SCs still go undetected attributable, in large part, to the non-disclosure of symptoms by a significant proportion of athletes from various backgrounds.^2–5^

From a public health perspective, symptom non-disclosure by athletes is critical considering that current concussion prevention and management protocols require athletes to cease participation when a concussion is suspected.^1^ Playing while symptomatic can prolong recovery and increase the risk of sustaining another concussion.^6^ The omission to disclose concussion symptoms also means that the injury remains undocumented, which puts the athlete at higher risk of accumulating traumatic brain injuries (TBIs) while still recovering from an initial concussion. Multiple studies have associated an accumulation of TBIs, especially in close temporal proximity, with more complicated clinical recovery and the development of persistent and debilitating symptoms in the long term.^7–12^

Symptom disclosure must be facilitated to optimize concussion management and safe return to play. To do so, it is necessary to identify and characterize factors influencing disclosure and explain their role within this decision-making process. The extant literature highlights three main reasons why athletes do not disclose their concussion symptoms: 1) not wanting to miss game time; 2) not wanting to let their team down; and 3) not thinking the injury was serious enough.^2,13,14^

Despite the important contributions of the aforementioned studies, gaps remain in our understanding of an athlete's disclosure decision making given that most studies used surveys that consisted of pre-selected and closed-ended questions. This approach prevents important athlete-generated insights that could deepen our understanding of the factors contributing to SC disclosure and describe their underlying motivations. This is consistent with the phenomenon of “satisficing” known to affect the quality of responses to surveys.^15^ This means that respondents tend to invest the minimal satisfying effort when completing a task rather than reflecting and thinking through every possible answer. In the concussion disclosure literature, satisficing manifests itself in the very rare use of the option “other” or by the selection of a single justification when more than one could apply.^2,16^ Therefore, studies using questionnaires can identify some of the contributing factors, but are ill-suited to describe how they may contribute to the decision-making process.

One way to overcome these limitations is to use qualitative methods of data collection and analysis. They are less prone to satisficing and can produce rich, participant-generated descriptions of events that are better suited for the investigation of multi-factorial and interactive processes underlying concussion disclosure.^17,18^ Thanks to their flexible data collection and the depth of their analysis, qualitative approaches have the potential to explore determinants of disclosure overlooked by quantitative studies (i.e., expanding our knowledge of which factors are relevant to SC disclosure) and provide the freedom to explore their effects on SC reporting (i.e., describing how each factor contributes to the decision-making process and their interactions).

Qualitative investigations have already revealed additional determinants of SC disclosure, such as the role of sympathetic arousal when decisions occur during competitions, the role of sociocultural expectations around sport injuries or the effect of the athlete's status within a team.^19–21^ They can also provide more details regarding the effects of known determinants of SC disclosure. For example, participants in one study described the concept of severe symptoms as those forbidding physical activity (i.e., loss of consciousness [LOC], strong nausea, dizziness, etc.), whereas those that can be played through (i.e., headache) would not warrant reporting.^22^

Qualitative research on concussion under-reporting remains rare and not without limitations. First, the interviews are often too structured and are guided by directed questions that come from surveys used in previous studies. In other words, the interviews lean toward known intrapersonal drivers of disclosure rather than trying to uncover novel and participant-generated insights. This leads into a second limitation. Most studies neglect to systematically distinguish between intrapersonal determinants (i.e., intrinsic factors specific to each athlete) and extrapersonal determinants (i.e., contextual factors affecting many athletes at once) of SC reporting. Third, the description of the major motives underlying SC disclosure remains siloed. Too rarely is there an attempt at integrating the SC reporting determinants into a holistic model highlighting their interactions and combined impact on the decision-making process.^20–22^ However, several important questions remain: 1) Have all relevant factors to concussion disclosure been uncovered?; 2) How do they interact to influence concussion disclosure?; and 3) What are the distinct contributions of intrapersonal factors?

Therefore, using semistructured interviews guided by grounded theory, the objective of this study is to explore and identify relevant intrapersonnel factors influencing SC disclosure and highlight their specific contribution to the decision-making process underlying reporting behaviors.

## Methods

### Design

Given this study's objective, semistructured interviews with constant comparative analysis, inspired from Straussian grounded theory (SGT), was identified as the most appropriate qualitative approach.^17^

### Ethics and demographics

Upon approval of this research project by the authors' institutional ethics committee, student-athletes from a Quebec-based university were solicited to participate into a study investigating the decision-making process underlying concussion disclosure. Recruitment occurred using a mix of criterion, purposive, and convenience sampling. The first author sent information about the study aims and design to the university's athletic department, which then relayed the information to the student-athletes competing in sports with known risks of concussion (i.e., cheerleading, rugby, and soccer) to ensure that we would interview persons with relevant knowledge and experience to our process of interest. Participants without a history of concussion (i.e., who reported having never experienced a concussion) were purposefully encouraged to participate and included in our sample because their perspective is relevant to the understanding of concussion reporting and is absent from the current literature on the topic.^22^

A total of 9 university student-athletes (see Table 1; 5 females, 4 males), representing three sports (soccer, rugby, and cheerleading), answered our solicitation by expressing their interest to the research team by e-mail and consented to being interviewed for this study. At the time of interview, none of the athletes with a history of concussion (HoC) was following a concussion recovery protocol. To preserve confidentiality, pseudonyms are used when presenting quotations from the participants.

**Table 1.** Participant Information

Participants	University sport	Age (years)	History of concussion (self-reported)
Anthony	Soccer	23.17	10
Bianca	Rugby	24.22	3
Hannah	Cheerleading	26.6	3
Inna	Cheerleading	21.6	3
Frank	Soccer	22.12	1
Charles	Soccer	24.66	0
Darya	Cheerleading	25.17	0
Evelyn	Rugby	19.87	0
Georges	Soccer	18.67	0

### Data collection and procedures

Interested participants who reached out to the research team received more detailed information regarding the study's design, and, after obtaining informed consent, a meeting was scheduled to conduct the interview. Before the first interview, the authors designed an open-ended guide to conduct in-depth, semistructured interviews. The first part of the interview gathered information on participants' HoC (when applicable) and general knowledge of concussion. Next, participants were asked to recall their most recent concussion and discuss their reporting process (e.g., Did they disclose their symptoms, yes or no?; Why or why not?; How and to whom?; What was the context?; etc.). For participants who reported no previous concussion, questions were asked in a hypothetical form (e.g., What would you do if you were to get a concussion next season?). Finally, some questions to allow exploration of the components of the Theory of Planned Behavior (i.e., attitudes, perceived subjective norms, and self-efficacy) were included in the interview guide given that they would be considered intrapersonal characteristics and had been previously reported as moderate influencers of concussion-reporting behaviors in athletes.^23,24^

In line with SGT procedures, analysis began after the first interview and proceeded in an iterative fashion, following principles of theoretical sampling.^17^ Concretely, it means that data collection and analysis occurred in parallel, and the interview guide was refined after each interview to accommodate purposeful exploration of novel concepts or gather more data on concepts and categories requiring more saturation. To respect the participant's availabilities, analysis of the previous interview was not always possible before the subsequent one. In such cases, the interviewer listened to the audio files shortly before the next interview, paying close attention to any relevant new insights and concepts to be incorporated within the interview guide. Examples of topics that were added to the interview guide after the first few discussions include: 1) professional sport ambitions and 2) role of culture and religion.

Data collection took place between May and August 2020, amid the COVID-19 pandemic. Nine semistructured interviews (range = 56–79 min; total = 587 min) were conducted remotely and recorded using Zoom software.^25^ Although it may seem like a low number of participants, qualitative studies are frequently published with lower numbers of either total number of participants or total minutes of interviews.^22,26^ Instead, constant comparison analysis inspired from grounded theory is more interested in data quality than quantity. Achieving conceptual saturation, meaning sufficient data collected to properly define and describe concepts and categories, is considered a good measure of data collection quality.^17,27^ Hence, recruitment stopped after the ninth interview, given that no new concepts had emerged from the last two interviews and that most concepts were deemed sufficiently saturated by the research team. To maximize conceptual saturation after the final interview, e-mails were sent to all previous participants to provide them with an opportunity to comment on concepts and categories that emerged after their interview.

All interviews were conducted, transcribed, and analysed by the first author. Save for one, all interviews were conducted in French. Citations were translated to English by the first author and reviewed by the second author.

### Data analysis

Verbatims of the interviews were uploaded, coded, and analyzed using QDAMiner software.^28^ Analysis followed procedures for constant comparative analysis inspired by grounded theory developed by Strauss and Corbin.^17^

The first step of analysis, known as open coding or line-by-line analysis, involved analyzing interview transcripts, sentence by sentence, to extract concepts. Concepts can be a word or short phrase representing a first-level abstraction of the meaning of the qualitative data. Extracted concepts are what guide the adjustments to the interview guide in the process of theoretical sampling referred above. Each new concept is constantly compared with those already identified to refine their definition and meaning. In the context of this study, concepts represent contributors to SC disclosure.

Then, when sufficient data were gathered to produce a significant number of well-defined concepts, the second step of analysis consisted of being grouped into a second level of abstraction called categories. Categories can then be described in terms of their properties (i.e., the concepts composing it) and dimensions (i.e., variations in its properties).

The final step of analysis, known as axial coding, occurred once several categories had emerged from the data and consisted in reanalyzing the original raw data to establish links between the categories and the concepts supporting them. In line with SGT, these links were established by exploring data for context, initial conditions, and action and interaction strategies and their consequences.^17^ These links were supported by data and by observations and reflections of the researcher, described and archived into written memos.^29^

In addition, extra steps were taken by the authors to improve the quality and credibility of the analytical process. A random sample (10%) of all codes were counter-coded by a non-author collaborator and achieved a concordance level of 87%. Further, the co-author and colleagues also acted as critics and provided feedback into the definition, description, and relationship among the concepts and categories.

## Results

After data analysis, 13 concepts contributing to concussion reporting emerged, across three intrapersonal categories: subjective evaluation of the concussion; concussion knowledge; and attitudes and behaviors. Descriptive summaries for each factor are presented in Table 2.

**Table 2.** Intrapersonal Factors and Categories Influencing Concussion Disclosure

Categories	Concepts	Description
Attitudes and behaviors	Intellectual vs. athletic prioritization	Valuing intellectual goals/activities over athletic ones would tend to favor disclosure. Vice versa.
	Role/status on the team	Athletes whose status on a team is less secure (i.e., rookies) or those who would perceive their role on the team to be either the physically tough player or that of a leader would be less likely to disclose than more secure peers.
	Maturity level	Described as the willpower to prioritize one's health and resist external influences, a high maturity level seems to favor disclosure.
	Tendency to play through injuries	Hiding and playing through injuries favors non-disclosure and all participants mentioned that it was a “default mode” for many athletes.
	Minimizing concussion-related risks	Athletes who tend to believe that risks of sustaining another concussion are low or that, if sustained, health risks and consequences of concussion are mostly trivial would be less likely to disclose them than their peers who perceive higher risks of sustaining a concussion or its future consequences.
Concussion knowledge	Factual knowledge	Capacity to recognize signs, symptoms, potential sequelae, and what to do if suspecting a concussion. Necessary but insufficient to favor disclosure.
	Experiential knowledge	Sum of an athlete's personal experience with concussions. Athletes without a history of concussion (HoC) would be much more likely to disclose their injury than athletes with an HoC. Also, athletes with a history of concussion would tend to use their worst experience as a benchmark against which future concussions are compared.
	Cultural knowledge	Athletes from countries/regions where social norms regarding traumatic brain injuries are more liberal than North America would be less likely to disclose concussions.
Subjective evaluation of concussion	Number	Number of concussion symptoms felt by an athlete. Greater number seems to favor disclosure.
	Intensity	Intensity of the symptoms felt by an athlete. Greater intensity would favor disclosure.
	Duration	Time frame during which an athlete feels/felt symptoms. Likelihood of disclosure seems to increase as duration increases.
	Visibility	Disclosure seems more likely for athletes whose symptoms are visible by others (e.g., nausea/vomiting) whereas disclosure would be less likely if symptoms felt by athletes are less visible to others (e.g., headache).
	Impairment of athletic capacities	Disclosure seems more likely when athletes are affected by symptoms directly impeding on athletic capacities (e.g., motor or vestibular deficits, vision impairments).

### Subjective evaluation of the concussion

Most athletes, regardless of concussion history status, mentioned that overall severity of their condition is the most important factor motivating their decision to disclose. They had been or would be more inclined to disclose symptoms if they were numerous, intense, of longer duration, visible to others, or significantly affected their athletic capacities. For example, sensory-motor manifestations like vestibular, visual impairments or LOC would incentivize athletes to disclose because they impact athletic performance and are highly visible. Inversely, invisible symptoms such as nausea or headaches, although uncomfortable, were considered easier to play through and less likely to promote disclosure.

*I believe the most important factor would be the severity of the symptoms right after the hit. If the symptoms are not too bad, if I'm capable of finishing the game despite the symptoms, I will.* – Charles

*[…] It also depends on the symptoms. If you have severe ones or more apparent ones, you'll be more inclined to disclose them. But if I'm unsure or I don't have that many or they're not visible, then I won't be inclined to tell my [athletic] trainer.* – Bianca

### Concussion knowledge

Athletes' decision to report concussion symptoms seems to be highly influenced by their knowledge of the injury. Our data suggest that this overall knowledge is made of three subtypes of knowledge: factual, experiential, and cultural. Factual knowledge would represent the capacity to recognize signs, symptoms, potential sequelae, and the procedures associated with concussion disclosure and management protocols. Experiential knowledge represents the personal experience of an athlete with concussions. This includes their experience with the injury (i.e., HoC), but also with concussion protocols and recovery process. Last, cultural knowledge refers to their social exposure to concussions and the norms surrounding their management within the context where they grew up or compete athletically.

From our data, factual knowledge seems necessary, yet insufficient for disclosure:

*Being knowledgeable about the signs and symptoms is important to at least being able to recognize a concussion. But it is really hard to do the first time you get a concussion, and I don't think it makes that much of a difference in the end.* – Anthony

*[…] most athletes I know are capable of recognizing symptoms of a concussion and many still do not disclose them.* – Bianca

On the other hand, experiential knowledge was reported as being highly influential by most participants. Athletes unanimously asserted that they reported or would report their first concussion owing to feelings of uncertainty and of the unknown:

*Because it was my first concussion, I told myself: “Better go get checked out to find out what this is.”* – Anthony

However, their decision to disclose subsequent concussions would depend on the comparison between their current concussion's severity and their previous ones:

*Now, if I get hit, I'll compare it to previous ones. Most often, I'll compare it to my worst one. If it is not as bad, I won't go see the doctor. But if it is worst, then I'll tell someone.* – Anthony

Finally, cultural differences in social norms regarding TBIs affect an athlete's capacity to recognize and report them. This factor is likely more relevant for athletes who come from environments where concussion management is more liberal:

*I'm not sure I would know how to tell if I had a concussion or even what to say. I only heard about concussions in Canada. In my native country, we never talked about it; when people hit their head, they just iced it and would be told to come back tomorrow.* – Frank

### Attitudes and behaviors

The category attitudes and behaviors contains five factors that seem to shape athletes' overall inclination toward concussion reporting: intellectual vs. athletic prioritization; role/status on the team; maturity level; tendency to play through injuries; and minimizing concussion-related risks.

First, prioritizing athletic goals seems to favor nondisclosure given that disclosure is likely to result in a short-term loss of playing time and could jeopardize longer-term objectives of a professional sport career by being labeled as having suffered several concussions or by missing opportunities to play in front of recruiters.

*A player for whom it is “all about sport,” well maybe being [more informed] won't change much because they might not need their head as much in the future. Maybe they don't care as much about the consequences.* – Georges

Conversely, giving higher priority to academics or intellectual work seems to favor genuine reporting:

*A friend of mine told me that he considered lying to his coaches, telling them he feels fine and that his symptoms are gone; but because he still had a lot of difficulty concentrating in school, he decided against it.* – Bianca

Second, participants reported that athletes whose status on the team is uncertain, such as rookies, are at high risk of under-reporting injuries, including concussions, for fear of losing their spot on the team:

*Take me for example. I'm a rookie at university this year and if I were to get injured, I would not want to say it.* – Evelyn

*For rookies, I believe the biggest fear is to lose your place on the team. Because if you get a concussion and have to miss a few weeks, they'll have to replace you in the meantime and there is a big chance that when you are ready to come back, they'll keep you on the bench.* – Inna

Additionally, those who would perceive their role on the team to be either physically tough or that of a leader would be less likely to disclose:

*If you identify as a leader on the team or have a certain image of “toughness” within the team, then you'd probably want to avoid letting your teammates down and break that image.* – Anthony

Third, most participants believed that being more mature correlates with concussion disclosure. They described maturity as the willpower to resist external influences. For example, prioritizing one's health over social reputation was considered a demonstration of maturity:

*I have enough maturity to understand that at some point my health is more important than a competition. While a less mature athlete might not have an appropriate threshold and might want to maintain a reputation of “toughness,” […] they might choose to risk their health to preserve their self-esteem.* – Evelyn

Fourth, all participants mentioned that hiding and playing through injuries was the “default mode” for many athletes:

*I've seen many stay on the mat despite having their bone almost coming out of their leg. We believe we have to stay unless we are literally about to die.* – Inna

Finally, athletes who minimize concussion-related risks would be more likely to under-report concussive injuries in the future:

*Our competitions are just two and half minutes and it is complicated to find a last-minute replacement for a cheer competition. So, yeah, I would most likely not disclose my symptoms and still compete.* – Darya

## Discussion

Our findings highlight the complex and multi-factorial nature of the decision-making process leading to SC disclosure. In our sample, we reported and explored 13 intrapersonal concepts influencing disclosure decisions across three categories: subjective evaluation of the concussion; concussion knowledge; and attitudes and behaviors.

Our results suggest that when sustaining a concussion, athletes tend to consider the severity of their injury as the starting point of their disclosure decision-making process. This pre-supposes sufficient factual knowledge for recognition of the concussion symptoms on the part of the athlete. Participants from our sample and data from other research both suggest that sufficient knowledge for recognition and subsequent disclosure is common for most university athletes in a North American context, but maybe less so for other populations.^4,14,30,31^ In the case of a concussion-naïve athlete sustaining their first concussion, disclosure would occur if the concussion severity was deemed higher than their personal tolerance threshold. An athlete's personal tolerance threshold would be determined and modulated by the concepts explored within the attitudes and behavior category, thereby generating interindividual variance regarding disclosure tendencies among athletes.

Further, our data suggest that concussion-naïve athletes tend to have a bias toward disclosure, most likely attributable to the shroud of unknown and uncertainty surrounding the injury lowering their personal tolerance threshold for disclosure. However, the experiential knowledge gained through this first concussion seems to significantly raise their disclosure threshold to the point of shifting their bias toward non-disclosure. Given that a large majority of athletes recover fully from their SC within 14 days,^32^ this shift may occur through dissipation of their fears regarding the health consequences of concussions and the development of an aversion toward the restriction of their daily activities while on concussion management protocols.

From this point on, our findings indicate that athletes with an HoC would adopt a non-disclosure bias and use their worst concussion experience as their new personal disclosure benchmark against which future concussions are compared. As summarized by our participant Anthony, they would consider disclosing a subsequent concussion only if they judged it to be more severe than their worst experience to date. Once again, concepts within the attitudes and behavior category would contribute to the decision-making process and generate interindividual variability by modulating adoption of the non-disclosure bias and the comparative evaluation of current and past concussions. If true, this mechanism would be quite concerning given that, as they accumulate experiential knowledge, athletes' disclosure threshold could likely heighten, crystalizing this non-disclosure bias.

Our description of the development of a non-disclosure bias subsequent to acquisition of a concussion history is an important contribution of this work and makes several links with the existing literature on the topic. This shift from a disclosure bias to a non-disclosure bias once athletes experience a concussion for the first time suggests that experiential knowledge may, in a population with a high level of awareness regarding concussions, bear more weight on disclosure decision making than factual knowledge. This is in line with surveys concluding that factual concussion knowledge is no longer a barrier to disclosure,^13,14,33^ and with recent studies revealing that an HoC is associated with a 2-fold increase in non-disclosure tendencies.^13,34^

Additionally, whereas the fact that athletes compare their current and previous concussions was suggested in a recent study,^22^ our data, such as Anthony's account, explicitly describe the mechanistic interaction between an athlete's experiential knowledge and their subjective evaluation of the current concussion symptoms.

In line with previous work, athletes in our sample also explained that a concussion is judged as severe when symptoms are of high intensity, of long duration, visible to others, or significantly impact their athletic capacities.^16,22^ In other words, athletes appear reluctant to report their symptoms unless they pose immediate health concerns (e.g., visual impairment) or are impossible to hide (e.g., LOC). However, at that point, one could argue that the decision to disclose is no longer made by them, but rather for them by the overt manifestations of the injury. Moreover, it appears that severity of the concussion can play a crucial role in potentially over-ruling the non-disclosure bias. This could explain why “judging the injury as not serious enough” is frequently reported by athletes as one of the key motives for non-disclosure.^2,14,22^ Given that practically all the studies on concussion reporting were conducted with athletes having an HoC, it is likely that the phrase “not serious enough” meant that their current concussion had failed to match the severity of their previous worst concussion and therefore insufficient to overturn their non-disclosure default behavior.

Another important contribution of our work is that it expands our knowledge of known determinants of SC disclosure, and it explored, to this day, unknown contributors to SC disclosure. To our knowledge, our work is the first to break down the influence of concussion knowledge into three subtypes (i.e., factual, experiential, and cultural). In previous studies, the term concussion knowledge was used in a way that would be equivalent to our concept of factual knowledge: knowledge of the most common concussion signs and symptoms, situations where concussions occur, and details about concussion management protocols.^16,22,35^ Though the effect of an HoC and of cultural differences in awareness of the injury on reporting tendencies had already been documented,^13,30^ our results suggest that they contribute to SC disclosure decision making by complementing athletes' factual knowledge and play a critical role (especially experiential knowledge) in establishing a non-disclosure bias.

Additionally, our observations that rookie athletes have a greater tendency to under-report concussions expands and refines the concept that certain athletes could be prone to under-reporting based on their status or role on the team.^21^ Meanwhile, the influence of factors, such as minimizing concussion consequences and tendency to play through injuries, could reflect expressions of “warrior mentality” or norms of “masculinity,” which have been known to act against symptom reporting.^19,21,36^

Our findings also include novel contributors to SC disclosure within the attitude and behaviors category such as prioritization of athletic versus intellectual activities and maturity level. Although they would benefit from further investigation and characterization in more diverse population samples, their presence in our results highlights some of the benefits and possible contributions of investigating concussion reporting by using qualitative methods to gather participant-generated insights.

Concepts under the category attitudes and behaviors seem to contribute to SC disclosure by determining athletes' personal disclosure threshold and, concurrently, modulating the development of a non-disclosure bias after a first concussion experience. This suggests that part of the interindividual differences in concussion disclosure tendencies among athletes would stem from differences in personality.^37^ A higher maturity level (i.e., capacity to resist external influences), a more secure status on their team, and prioritizing intellectual activities could lower an athlete's personal concussion severity threshold. Conversely, tendencies to play through injuries, minimizing concussion-related risks, favoring athletic pursuits above all else, and an insecure status on the team could raise an athlete's personal disclosure threshold.

### Limitations

Although in-depth interviews were conducted, our sample could benefit from more diversity in age and sports affiliation to verify whether our results can be transferred to other age groups, levels of competition, and sports. For example, given that factors like status on the team influence disclosure, future work should also gather the point of view of athletes practicing individual sports. Also, given that cultural knowledge, maturity level, and minimizing concussion-related risks are novel concepts, further research is likely required to investigate them more thoroughly.

### Implications for concussion education and awareness programs

Current concussion education programs focus mostly on teaching factual knowledge (i.e., recognizing signs and symptoms, health risks, proper management, etc.) and have had limited success in improving disclosure.^38^ A recent study even observed a significant decline in concussion disclosure after an improvement in concussion knowledge.^16^

As illustrated by another recent study, concussion disclosure rises with increasing level of awareness among its population.^30^ However, that does not mean that concussion education and factual knowledge are not important. By subdividing concussion knowledge into three distinctive components (i.e., factual, experiential, and cultural), our work proposes an explanation to reconcile this seemingly contradictory effect of concussion education. It suggests that educational programs focusing on factual concussion knowledge can facilitate disclosure if it provides athletes with the ability to recognize their symptoms and the steps to follow to disclose them for maximum health benefits. Such programs would be warranted in contexts and populations where concussion awareness is known to be low or inadequate. However, it also suggests that, in contexts and populations where most athletes already possess this knowledge (e.g., Western/North American university athletes), these education programs may have reached a ceiling given that the maximum benefits of factual knowledge in terms of helping athletes recognize concussion symptoms have already been achieved. Further, based on the other forms of concussion knowledge emerging from our data (i.e., cultural and experiential), we hypothesize that continuous focus on factual knowledge in such a context may lead to a reduction in disclosure by providing athletes with additional tools to “game” concussion protocols and recovery.

Hence, to further improve disclosure, we could attempt to reframe and reverse an athlete's non-disclosure “default mode.” To do so, our data suggest that a set of specific intrapersonal factors must be addressed. At an individual level, this could be done by 1) enabling an athlete to put their own concussion experience within the perspective of long-term health risks; 2) encouraging prioritization of intellectual over athletic activities; and 3) by interventions aimed at fostering maturity. At an organizational level, coaches and teams could help by rewarding health-promoting behaviors over performance-driven behaviors (e.g., playing through injuries) and reassuring athletes about their status on the team, through proactive, open, and transparent communication of expectations. Future research should verify whether these proposed interventions successfully reframe an athlete's personal non-disclosure default mode and translate into greater concussion disclosure tendencies in different athlete populations.

## Conclusion

Our work highlights the benefits of using a qualitative approach to study the multi-factorial processes underlying concussion disclosure. Thanks to in-depth interviews, our work identified novel variables relevant to disclosure decision making that had not been revealed by questionnaires and surveys. Using grounded theory, we were also able to describe how athletes with an HoC could be prone to adopt a default non-disclosure bias. This bias seems to be driven mostly by comparing the severity of a current concussion to their personal worst concussion. It is also weighted by intrapersonal factors such as cultural knowledge of concussions, status on the team, maturity level, and conflicting priorities between athletic and intellectual activities.

If echoed into a larger population, our results would suggest that concussion education programs working toward reframing and reversing athletes' non-disclosure bias could enhance an athlete's disclosure tendencies. Individualized interventions based on personal characteristics of athletes, such as concussion history, tendencies to play through injuries, and insecurities about status on the team or maturity level, could yield more benefits than pursuing the status quo.

## Transparency, Rigor, and Reproducibility Summary

Our exploratory qualitative approach used a mix of criterion and purposive sampling. Criterion sampling took place by recruitment of student-athletes competing in sports with known risks of concussion (i.e., rugby [male and female], soccer [male and female], or cheerleading [co-ed]) whereas purposive sampling refers to our intentional pre-data collection decision to include participants who did not have an HoC. A total of 9 university student-athletes (5 females, 4 males) representing three sports (soccer, rugby, and cheerleading) answered our call for participants and consented to being interviewed. Data collection and analysis followed SGT with epistemological roots in critical realism and symbolic interactionism. Before the first interview, the authors designed an open-ended interview guide to conduct in-depth, semistructured interviews. The initial interview guide was designed with both the aims of the research project as well as summary knowledge of the current literature on concussion reporting. The interview guide was modified along the data collection process using theoretical sampling. Data collection took place between May and August 2020, amid the COVID-19 pandemic. Interviews lasted between 56 and 79 min and were conducted remotely and recorded using Zoom software.

All interviews were conducted by the first author. After the eighth interview, no new major concept or insight emerged. A final interview was scheduled to confirm this trend and bonify descriptions of certain concepts and categories to reach saturation. The first 7 participants interviewed were given an opportunity to comment (orally or in writing) on the new concepts that emerged in the interviews that occurred after theirs. Two of them responded, and their answers were included in the final rounds of analysis and during the conception of the theory. All interviews were conducted and transcribed verbatim, by the first author. Analysis began after the first interview and proceeded in an iterative fashion to inform future data collection (i.e., theoretical sampling). Coding and analysis were done using QDAMiner software. A random sample (i.e., 10%) of all codes were counter-coded by a non-author collaborator, and a concordance level of 87% was achieved. For the few cases where there was disagreement, short discussions allowed to modify certain code attributions or refine the definition of certain concepts. The co-author and non-author colleagues also acted as critics and provided feedback into the definition, description, and relationship of the concepts and categories.

Finally, a more comprehensive review of the concussion reporting literature was performed after completion of the data analysis to enhance and refine the generated grounded theory. Both the initial and final interview guides can be provided upon request, as well as the verbatims, codes, and memos produced during analysis, and theoretical integration can be shared upon request.

## Abbreviations Used

HoC: history of concussion
LOC: loss of consciousness
SC: sports concussion
SGT: Straussian grounded theory
TBI: traumatic brain injury
